# Differentially Regulated Transcription Factors and ABC Transporters in a Mitochondrial Dynamics Mutant Can Alter Azole Susceptibility of *Aspergillus fumigatus*

**DOI:** 10.3389/fmicb.2020.01017

**Published:** 2020-05-26

**Authors:** Laura Sturm, Bernadette Geißel, Ronny Martin, Johannes Wagener

**Affiliations:** ^1^Max von Pettenkofer-Institut für Hygiene und Medizinische Mikrobiologie, Medizinische Fakultät, LMU München, Munich, Germany; ^2^Institut für Hygiene und Mikrobiologie, Julius-Maximilians-Universität Würzburg, Würzburg, Germany; ^3^National Reference Center for Invasive Fungal Infections (NRZMyk), Jena, Germany

**Keywords:** *Aspergillus fumigatus*, mitochondrial dynamics, azole resistance, efflux pumps, transcription factors

## Abstract

Azole resistance of the fungal pathogen *Aspergillus fumigatus* is an emerging problem. To identify novel mechanisms that could mediate azole resistance in *A. fumigatus*, we analyzed the transcriptome of a mitochondrial fission/fusion mutant that exhibits increased azole tolerance. Approximately 12% of the annotated genes are differentially regulated in this strain. This comprises upregulation of Cyp51A, the azole target structure, upregulation of ATP-binding cassette (ABC) superfamily and major facilitator superfamily (MFS) transporters and differential regulation of transcription factors. To study their impact on azole tolerance, conditional mutants were constructed of seven ABC transporters and 17 transcription factors. Under repressed conditions, growth rates and azole susceptibility of the mutants were similar to wild type. Under induced conditions, several transcription factor mutants showed growth phenotypes. In addition, four ABC transporter mutants and seven transcription factor mutants exhibited altered azole susceptibility. However, deletion of individual identified ABC transporters and transcription factors did not affect the increased azole tolerance of the fission/fusion mutant. Our results revealed the ability of multiple ABC transporters and transcription factors to modulate the azole susceptibility of *A. fumigatus* and support a model where mitochondrial dysfunctions trigger a drug resistance network that mediates azole tolerance of this mold.

## Introduction

*Aspergillus fumigatus* is an opportunistic fungal pathogen that causes life-threatening airborne infections in immunocompromised patients. The disease is called invasive aspergillosis and associated with high mortality rates of up to 95% (Kousha et al., 2011; Brown et al., 2012; Kosmidis and Denning, 2015). Treatment of invasive aspergillosis relies on the administration of antifungals. The azole class of antifungals is currently recommended as first line treatment for infections caused by *A. fumigatus* (Patterson et al., 2016). Azoles target the ergosterol biosynthesis pathway by inhibiting the lanosterol 14α-demethylase (CYP51) which is fungicidal for the mold *A. fumigatus*. However, in the recent years azole-resistant *Aspergillus* strains are emerging, thereby challenging the efficacy of current azole-based therapies (van der Linden et al., 2015; Perlin et al., 2017). As such, azole resistance was associated with therapy failure and increased mortality in patients suffering from invasive aspergillosis (van der Linden et al., 2015; Lestrade et al., 2019).

The majority of the azole-resistant clinical *A. fumigatus* isolates harbor resistance-mediating mutations in the target enzyme, the lanosterol 14α-demethylase (CYP51, also known as Erg11), or in its promoter region (Dudakova et al., 2017; Perlin et al., 2017). Unfortunately, the mechanistic nature facilitating azole resistance of the remaining clinical isolates – which may represent up to 50% of the azole resistant clinical isolates (Fraczek et al., 2013) – is largely unknown. Azole resistance of *Candida* species is often mediated by increased expression of efflux pumps (Morschhäuser, 2016; Prasad et al., 2016). It was therefore proposed that upregulation of drug efflux could represent the second most abundant azole resistance mechanism in *A. fumigatus* (Cowen et al., 2015; Dudakova et al., 2017; Perlin et al., 2017). Indeed, increased expression of several efflux pumps was observed in several azole resistant clinical *A. fumigatus* isolates without CYP51 mutations (Fraczek et al., 2013). However, experimental evidence that clearly demonstrates overexpression of these or other efflux pumps causes azole resistance of *A. fumigatus* is still lacking (Fraczek et al., 2013; Moye-Rowley, 2015; Dudakova et al., 2017). So far, only two ATP-binding cassette (ABC) transporters were shown to affect the azole tolerance of the mold under *in vitro* conditions (nicely summarized in Moye-Rowley, 2015).

Recently, we characterized the role of the mitochondrial fusion and fission machinery for viability, virulence and antifungal drug susceptibility of *A. fumigatus* (Neubauer et al., 2015). Surprisingly, we found that the disruption of mitochondrial fission and of mitochondrial fission and fusion results in *Aspergillus* mutants that exhibit increased azole tolerance. A role of mitochondrial function in azole tolerance was previously reported for pathogenic and non-pathogenic yeasts and linked to altered expression of efflux pumps (Traven et al., 2001; Tsai et al., 2006; Ferrari et al., 2011a, b; Shingu-Vazquez and Traven, 2011; Peng et al., 2012). But the exact mechanism responsible for the increased azole tolerance remained unresolved. Here we show that the expression of multiple ABC superfamily and major facilitator superfamily (MFS) transporters is upregulated in the mitochondrial fission and fusion mutant. In addition, expression of *cyp51A*, the gene that encodes one of two CYP51 orthologs, was upregulated and the expression of several transcription factors were up- or downregulated. Construction and phenotypic characterization of conditional mutants of the differentially regulated genes revealed the ability of several of the identified transcription factors and ABC transporters to affect the azole susceptibility of *A. fumigatus*. Our results suggest that mitochondrial dysfunction triggers increased expression of CYP51 and induction of efflux pumps in *A. fumigatus* which could mediate the increased azole tolerance observed in such mutants.

## Materials and Methods

### Strains, Culture Conditions, and Chemicals

The non-homologous end joining-deficient *A. fumigatus* strain AfS35 was used as wild type in this study (Krappmann et al., 2006; Wagener et al., 2008). The conditional mutants *mdu1*_tetOn_ (AFUA 8G07000), *mdu2*_tetOn_ (AFUA_1G03800), *mdu3*_tetOn_ (AFUA_5G01650), *mdu4*_tetOn_ (AFUA_8G07280), *mdu5*_tetOn_ (AFUA_2G15340), *mdu6*_tetOn_ (AFUA_4G01470), *mdu7*_tetOn_ (AFUA_4G00710), *atfD*_tetOn_ (*mdu8*_tetOn_; AFUA_6G12150), *mdu9*_tetOn_ (AFUA_2G09330), *mdu10*_tetOn_ (AFUA_1G14860), *mdu11*_tetOn_ (AFUA_2G14350), *mdd1*_tetOn_ (AFUA_5G14290), *mdd2*_tetOn_ (AFUA_1G15910), *mdd3*_tetOn_ (AFUA_6G06535), *gliZ*_tetOn_ (*mdd4*_tetOn_; AFUA_6G09630), *mdd5*_tetOn_ (AFUA_6G12160), *mdd6*_tetOn_ (AFUA_4G06880), *abc1*_tetOn_ (AFUA_3G01400), *mdr1*_tetOn_ (*abc2*_tetOn_; AFUA_5G06070), *abc3*_tetOn_ (AFUA_6G08020), *abc4*_tetOn_ (AFUA_4G14130), *abc5*_tetOn_ (AFUA_6G03080), *abc6*_tetOn_ (AFUA_5G10510), and *abc7*_tetOn_ (AFUA_3G07300) were constructed by inserting a doxycycline-inducible promoter cassette (oliC-tetOn; pJW128) before the coding regions of the indicated genes, essentially as described before (Helmschrott et al., 2013). The deletion mutants Δ*mdu2*, Δ*mdu3*, Δ*mdu11*, Δ*mdd3*, Δ*gliZ* (Δ*mdd4*), Δ*mdd5*, Δ*abc1*, Δ*mdr1* (Δ*abc2*), Δ*abc3*, and Δ*abc4* in the parental strain Δ*dnm1 mgm1*_tetOn_ (Neubauer et al., 2015) were constructed using a phleomycin resistance cassette. The correct integration of the cassettes was verified using diagnostic PCRs, essentially as described before (Geißel et al., 2018). Conidia were harvested from cultures on Aspergillus minimal medium (AMM; Hill and Kafer, 2001) at 37°C. The conditional mutant *mdd6*_tetOn_ was always raised under induced conditions on medium supplemented with 0.5 μg ml^–1^ doxycycline. Experiments were performed on or in Sabouraud medium [4% (w/v) d-glucose, 1%(w/v) peptone (#LP0034; Thermo Fisher Scientific; Rockford, IL, United States, pH 7.0]. When indicated, medium was supplemented with doxycycline (#631311; Clontech; Mountain View, CA, United States) or voriconazole (#A4320; Apexbt Technology LLC; Houston, TX, United States). Solid medium was supplemented with 2% (w/v) agar (214030; BD, Franklin Lakes, NJ, United States). Etest strips were obtained from bioMérieux (Marcyl’Etoile, France). To fully induce the conditional Tet-On promoter, medium was supplemented with 15 μg ml^–1^ if not stated differently (Helmschrott et al., 2013; Dichtl et al., 2015; Loiko and Wagener, 2017). Broth microdilution experiments were performed in 96 well plates. To this end, conidia were inoculated in 100 μl medium per well at a concentration of 5 × 10^4^ conidia ml^–1^. Medium was supplemented with serial voriconazole dilutions (×0.75) starting with a concentration of 3 μg ml^–1^ voriconazole. Plates were incubated at 37°C and analyzed after 48 h.

### RNA Sequencing

For the gene expression analyses, three RNA samples were isolated and analyzed per strain (wild type and Δ*dnm1 mgm1_tetOn_*). To this end, 2.5 × 10^7^ conidia were inoculated in 50 ml Sabouraud liquid medium and cultured for 15 h at 37°C. Mycelium was separated from culture medium and washed with PBS buffer solution. RNA was extracted by pestling 100 mg mycelium in liquid nitrogen and immediately suspending the frozen ground powder in 450 μl RLT buffer (RNeasy Mini Kit (50); #142349288, QIAGEN GmbH, D-40724 Hilden). The RTL buffer suspension was subsequently processed according to the manufacturer’s instructions. RNA quality and quantity were analyzed with the Experion RNA StdSens Analysis Kit (#700-7103, Bio-Rad Laboratories, Hercules, CA, United States) and Experion System (100-240V, for RNA and DNA analyses, includes electrophoresis station, priming station, vortex station, software, USB2 cable, instructions) (#700-7001, Bio-Rad Laboratories, Hercules, California, United States). RNA sequencing was subsequently performed by GATC Biotech AG (InView Transcriptome Explorer; Konstanz, Germany) and the transcriptome mapped on the Af293 genome.

### Reverse Transcription–qPCR

Three RNA samples were isolated and analyzed per strain (wild type, *mdd3*_tetOn_ and *mdu2*_tetOn_) and condition (with and without 15 μg ml^–1^ doxycycline). To this end, 2.5 × 10^7^ conidia were inoculated in 50 ml Sabouraud liquid medium with and without doxycycline and cultured for 12 h at 37°C. Mycelium was separated from culture medium and disrupted in Lysing-Matrix-C tubes (M.P. Biomedical; Irvine, CA, United States) supplemented with TRIzol Reagent (#15596-026, ambion/RNA, life technologies; Carlsbad, CA, United States) in a FastPrep-24 homogenizer (M.P. Biomedical; at 6.5 m s^–1^ for 60 s followed by cooling in an ice-cold water bath). RNA was subsequently purified following the instructions of the manufacturer of the TRIzol Reagent. The RNA pellet was resuspended with 60 μl DEPC-treated water (#AM9922, Ambion, Thermo Fisher Scientific; Rockford, IL, United States). Gene expression was then quantified with 100 ng RNA per PCR (100 ng μl^–1^) and specific primers and the Luna Universal One-Step-RT-qPCR Kit (#E3005; New England Biolabs, Ipswich, MA, United States) in a qTower3 (Analytik Jena, Jena, Germany). Expression data was normalized to *act1* (actin; AFUA_6G04740) and analyzed with the ΔΔCt method described by Pfaffl (Pfaffl, 2001). Statistical analyses were performed with GraphPad Prism 5 (GraphPad Software, La Jolla, CA, United States). Statistical significance was calculated using ANOVA with *post hoc* Tukey.

### Transcriptome Analysis and Gene Ontology

RNA sequencing data were analyzed with Cuffdiff (Trapnell et al., 2010) using the web-based Galaxy platform (Afgan et al., 2018)^1^ to identify differentially expressed genes in the repressed Δ*dnm1 mgm1_tetOn_* mutant in comparison to wild type (*p* = 0.007). Further analyses were performed with Gene Ontology Enrichment (Ashburner et al., 2000) of FungiDB (Basenko et al., 2018) by searching for over- and underrepresented GO terms (Gene ontology enrichment^2^; goslim_generic subset; molecular function/biological process; *p* = 0.05; 06.03.2018). The following GO term annotations were examined separately: mitochondrion (GO:0005739), mitochondrion organization (GO:0007005), transmembrane transport (GO:0055085), transmembrane transporter activity (GO:0022857), response to stress (GO:0006950), ergosterol biosynthetic process (GO:0006696), and regulation of ergosterol biosynthetic process (GO:0032443) (FungiDB; my strategies/genes/function prediction/GO term; 20.03.2019). For identification of the ABC and MFS transporters as well as the transcription factors which are up- and downregulated in Δ*dnm1 mgm1_tetOn_* the differentially expressed genes were compared with the genes coding the indicated proteins using the free software Knime^3^.

### Database Searches and Sequence Analysis

To obtain DNA and protein sequences as well as ontology and protein function prediction the following databases were used for the respective species *A. fumigatus*: Aspergillus Genome Database^4^, Af293 (01.04.2016) (Cerqueira et al., 2014) and Fungi DB^2^, Af293 (28.03.2018) (Basenko et al., 2018); *S. cerevisiae*: Yeast Genome Database^5^, S288C (01.04.2016; 28.03.2018) (Engel et al., 2014); *Candida albicans*: Candida Genome Database^6^, SC5314/assembly 22 (28.03.2018) (Skrzypek et al., 2017). Pfam domains (PF00005^7^, IPR003439 ABC_tran ABC transporter-like; PF01061, IPR013525 ABC2_membrane ABC-2 type; PF14510, ABC transporter extracellular N- terminal; PF06422, IPR010929 PDR_CDR CDR ABC transporter; PF00664, IPR001140 ABC_membrane ABC transporter, transmembrane domain; PF06472, IPR010509 ABC_membrane_2 ABC transporter, N-terminal; PF12848, ABC_tran_2 ABC transporter; PF00385, Chromo (Chromatin Organization Modifier) domain; PF04068, IPR007209 RLI RNase L inhibitor RLI, possible metal-binding domain; PF00037, IPR001450 Fer4 4Fe-4S binding domain; PF12661, IPR013032 hEGF EGF-like, conserved site; PF12698, ABC2_membrane_3; PF02492, IPR003495 cobW CobW/HypB/UreG domain) (Finn et al., 2016) of all known ABC transporters of *S. cerevisiae* (Klein et al., 2011) were used to analyze the genome of *A. fumigatus* to identify and classify the 52 ABC transporters. A similar approach was used to identify and classify the 211 MFS transporter in the genome of *A. fumigatus* (Pfam domain PF07690 (IPR011701 MFS_1 Major facilitator superfamily; Costa et al., 2014; Dos Santos et al., 2014). Putative transcription factors were identified using Gene Ontology (GO:0006355 – regulation of transcription, DNA-templated) and further classified with correlating Pfam domains (PF07716, Basic region leucine zipper; PF00172, Fungal Zn(2)-Cys(6) binuclear cluster domain; PF04082, Fungal specific transcription factor domain; PF05920, Homeobox KN domain; PF00170, bZIP transcription factor; PF11951, Fungal specific transcription factor domain; PF13086, AAA domain; PF13087, AAA domain).

## Results

### Comparison of the Transcriptome of the *Δdnm1 mgm1_tetOn_* Mutant With the Wild Type Strain

**FIGURE 1:**
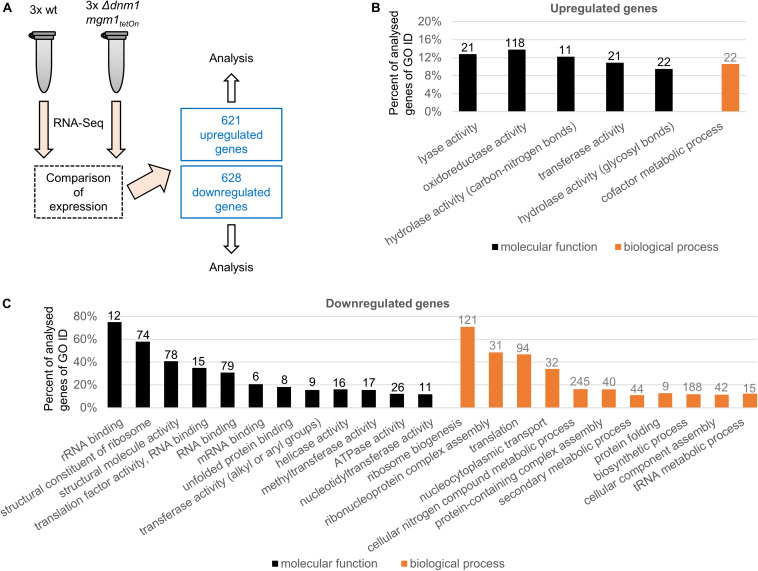
Analysis of differentially expressed genes in the Δ*dnm1 mgm1_tetOn_* mutant. **(A)** The transcriptome of wild type (wt) and Δ*dnm1 mgm1_tetOn_* were analyzed with RNA sequencing in triplicates. Differentially expressed genes were subsequently identified with Cuffdiff ^32^. 621 genes were upregulated and 628 genes were downregulated (*p* ≤ 0.007). **(B,C)** Significantly overrepresented **(B)** and underrepresented **(C)** GO terms (goslim_generic subset; *p*-value ≤ 0.05) for molecular function (black bars) and biological process (gray bars). The bars indicate the percentage of identified genes of all analyzed *A. fumigatus* genes associated with each GO slim term. The absolute number of identified genes is shown on top of each bar.

Mitochondria continuously undergo fission and fusion processes. Two distinct and partially conserved machineries accomplish mitochondrial fission and fusion (Pernas and Scorrano, 2016). We previously studied the fission and fusion machineries of *A. fumigatus* by constructing and characterized conditional and deletion mutants of several core components of the respective machineries (Neubauer et al., 2015; Wagener, 2016). Of those the conditional Δ*dnm1 mgm1_tetOn_* mutant was deleted for the mitochondrial fission dynamin Dnm1 (important for fission) whereas the mitochondrial fusion dynamin Mgm1 (important for fusion) is under control of a doxycycline-inducible Tet-On promoter. The Δ*dnm1 mgm1_tetOn_* mutant exhibits a remarkable azole tolerance under induced conditions, very similar to the fission single deletion mutant Δ*dnm1* or mutants lacking other key components of the mitochondrial fission machinery such as Δ*mdv1* and Δ*fis1*. Under repressed conditions, which means inactivation of fission and fusion, the azole tolerance is even further pronounced (Neubauer et al., 2015). To identify and explore potential mechanisms that contribute to the azole resistance of *A. fumigatus*, we compared the transcriptome of the Δ*dnm1 mgm1_tetOn_* mutant with the wild type. To this end, freshly harvested conidia were inoculated in Sabouraud medium and cultured for 15 h at 37°C. RNA was extracted and subjected to RNA sequencing and differential expression analysis. As shown in Figure 1A and Supplementary Tables S1–S3, 1249 genes – approximately 12% of the annotated *A. fumigatus* genome – were differentially expressed in the Δ*dnm1 mgm1_tetOn_* mutant under repressed conditions compared to wild type (*p* = 0.007). Of those, 621 genes were upregulated and 628 genes were downregulated.

As expected, the gene with the highest negative fold-change was *dnm1*, the gene deleted in the double mutant. Surprisingly, no repression of *mgm1* was detected even though the strain was cultured under repressive conditions (w/o doxycycline). To exclude mistaken identity of the analyzed strains we examined the RNA sequencing coverage (Supplementary Figure S1A). In contrast to the wild type where the *mgm1* coverage already starts in the 5′ untranslated region, coverage in the Δ*dnm1 mgm1_tetOn_* mutant begins with the start codon of *mgm1* which is in line with the integration of the Tet-On promoter at this site. Since the repressibility of the analyzed strain is evident based on distinct growth phenotypes under repressed and induced conditions (Neubauer et al., 2015 and Supplementary Figure S1B), we concluded that the non-detected differential expression results from a combination of the leakiness of the conditional promoter system and potential heterogeneous expression of *mgm1* in different vegetative/stationary hyphal compartments of the mycelium (Hewitt et al., 2016).

A genome ontology (GO) enrichment analysis was performed to identify which GO terms are over- and underrepresented (FungiDB database/gene ontology enrichment; goslim_generic subset; *p* = 0.05; Figures 1B,C; Ashburner et al., 2000; Basenko et al., 2018). Only, five molecular function and one biological process GO terms were overrepresented in the upregulated genes, those were primarily related to metabolism (Figure 1B and Supplementary Tables S4, S5). In contrast, multiple molecular function (12) and biological process (11) GO terms were enriched in the down regulated genes (Figure 1C and Supplementary Tables S6, S7). Interestingly, many of those (6 of 12 molecular function GO terms and 6 of 11 biological process GO terms) were related to ribosome and protein biogenesis.

**FIGURE 2:**
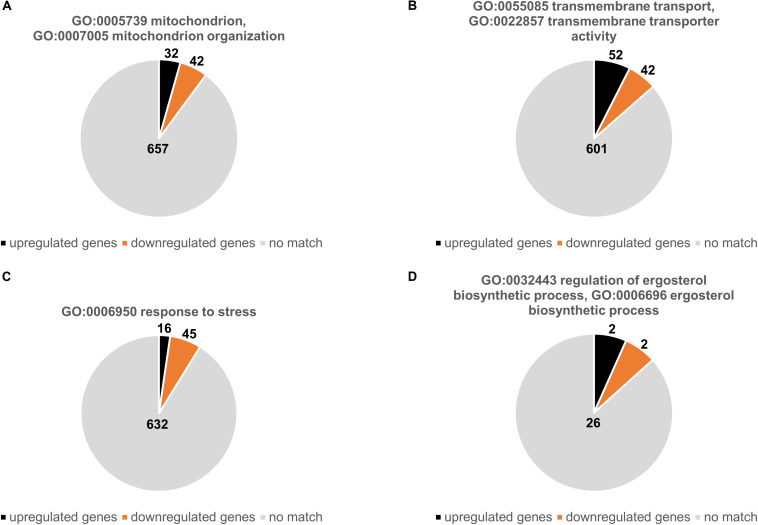
Up- and downregulated genes Δ*dnm1 mgm1_tetOn_* mutant with a presumed role in mitochondrial biology, transmembrane transport, stress response and ergosterol metabolism. The charts show the number of up- and downregulated genes that belong to the indicated GO terms: **(A)** mitochondrion (GO:0005739) and mitochondrion organization (GO:0007005); **(B)** transmembrane transport (GO:0055085) and transmembrane transporter activity (GO:0022857); **(C)** response to stress (GO:0006950); **(D)** ergosterol metabolic process (GO:0008204). The absolute number of genes associated with the indicated GO terms is shown in charts.

We subsequently analyzed the number of genes that were either up- or downregulated with respects to specific GO terms related mitochondrial functions and known azole resistance mechanisms (Figures 2A–D). Of 731 genes associated with the mitochondrion (GO:0005739) or with mitochondrion organization (GO:0007005) 32 were upregulated and 42 downregulated (Figure 2A and Supplementary Table S8). Of 693 genes that were associated with transmembrane transport (GO:0055085) and transmembrane transporter activity (GO:0022857) 52 were upregulated and 42 downregulated (Figure 2B and Supplementary Table S9). Interestingly, only 16 of 693 genes associated with response to stress (GO:0006950) were upregulated and 45 downregulated (Figure 2C and Supplementary Table S10). Of the 30 genes that were annotated for being involved in ergosterol biosynthetic process (GO:0006696) or regulation of ergosterol biosynthetic process (GO:0032443) only two were up- and two downregulated. The two upregulated genes were AFUA_1G04540 (1.5-fold) and AFUA_1G17190 (1.6-fold) which encode the homologs of the *Saccharomyces cerevisiae* proteins ScMcr1, a mitochondrial NADH-cytochrome b5 reductase involved in ergosterol biosynthesis (YKL150W), and Pcs60, an oxalyl-CoA synthetase (YBR222C), respectively. The two downregulated genes were AFUA_3G10660 (0.6-fold) and AFUA_7G01220 (0.7-fold) which encode the homologs of *S. cerevisiae* Erg13, the HMG-CoA synthase (YML126C), and Erg9, the squalene synthase (YHR190W) (Figure 2D and Supplementary Table S11). Notably, *cyp51A*, the drug target of azole antifungals was not annotated for being involved in the ergosterol biosynthetic process (GO:0006696), even though it was 1.6-fold upregulated in the Δ*dnm1 mgm1_tetOn_* mutant.

### Multiple Efflux Pumps Are Upregulated in the Mitochondrial Fission/Fusion Mutant

**FIGURE 3:**
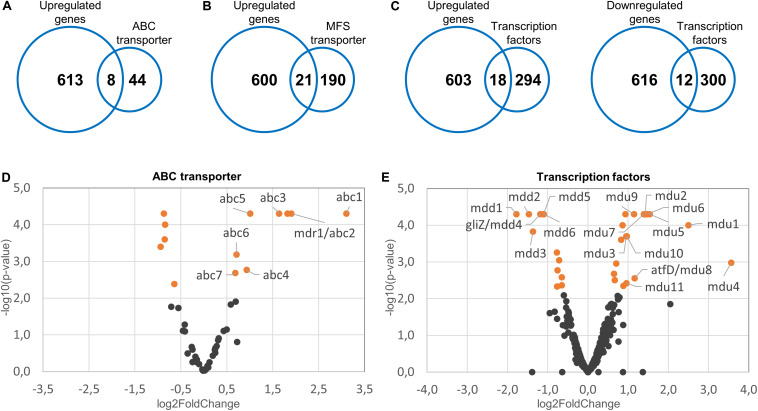
Upregulated efflux pumps and differentially regulated transcription factors in the Δ*dnm1 mgm1_tetOn_* mutant. Fifty-two ABC transporters, 211 MFS transporters and 312 transcription factors were identified in the reference genome of *A. fumigatus*. The identified genes were subsequently compared to the list of genes upregulated (*n* = 612) or downregulated (*n* = 628) in the Δ*dnm1 mgm1_tetOn_* mutant. The number of upregulated ABC transporters **(A)**, upregulated MFS transporters **(B)**, and up- or downregulated transcription factors **(C)** are indicated in the intersections of the Venn diagrams. **(D,E)** Volcano plots of the expressed ABC transporters **(D)** and transcription factors **(E)**. The log_2_ fold changes of the individual genes (x-axis) were plotted against the corresponding adjusted *p*-values (-log_10_; y-axis). Differentially expressed genes (*p* = 0.007) are indicated in orange. Genes that were further analyzed by constructing conditional mutants thereof were labeled accordingly.

Mitochondrial dysfunctions in baker’s yeast and in the opportunistic pathogen *Candida glabrata* cause the induction of a partially conserved pleiotropic drug resistance (PDR) network. This involves the increased expression of multiple efflux pumps (Traven et al., 2001; Tsai et al., 2006; Gulshan et al., 2008; Ferrari et al., 2009, 2011a,b; Shingu-Vazquez and Traven, 2011). We speculated that a similar mechanism exists in *A. fumigatus* which could explain the increased azole tolerance of the mitochondrial fission/fusion mutant. The genome of *A. fumigatus* encodes 52 ABC proteins and 211 MFS transporters, based on a Pfam database search for proteins annotated as ABC transporter (PF00005) or MFS (PF07690) (Supplementary Tables S12, S13). Of those, eight ABC proteins (15%) and 21 MFS transporters (10%) were upregulated in the fission/fusion mutant compared to wild type (Figures 3A,B and Supplementary Tables S14, S15). To our knowledge, none of these proteins has been characterized in *A. fumigatus* to date. A detailed analysis of the upregulated transporters revealed that several may have a role in drug efflux. One of the eight upregulated ABC proteins (encoded by Afu1g16440) belongs to the YEF3/RLi ABC protein family which is not implicated in transport and was therefore not considered as potential transporter. Four of the seven remaining upregulated putative ABC transporters have homologs in *C. albicans* or *S. cerevisiae* that were previously implicated in azole resistance (Supplementary Table S14). Similar, of the 21 upregulated MFS transporters nine have homologs in *C. albicans* or *S. cerevisiae* that were previously reported to mediate azole resistance (Supplementary Table S15).

### Differentially Regulated Transcription Factors in the Mitochondrial Fission/Fusion Mutant

Induction of the pleiotropic drug response network in baker’s yeast and *C. glabrata* depends on a conserved zinc finger transcription factor that was named *Sc*Pdr3 or *Cg*Pdr1, respectively (Shingu-Vazquez and Traven, 2011). Unfortunately, due to the high number of highly similar transcription factors present in other species, functional orthologs cannot be easily identified with conventional homology searches. The genome of *A. fumigatus* encodes more than 300 potential transcription factors (GO:0006355; regulation of transcription, DNA-templated; Supplementary Table S16). Of those, 120 exhibit the Pfam domains PF00172 [fungal Zn(2)-Cys(6) binuclear cluster domain] and PF04082 (fungal specific transcription factor domain) which are the only two common domains that can be identified in *Sc*Pdr3 and *Cg*Pdr1 (Supplementary Table S16).

Interestingly, *Sc*Pdr3 as well as *Cg*Pdr1 were reported to regulate their own expression (Delahodde et al., 1995; Paul et al., 2011). We speculated that similar transcription factors could be involved in the increased azole tolerance of the fission/fusion mutant and that such functional orthologs would then also autoregulate their own expression. As shown in Figure 3C of the 312 potential transcription factors 18 were upregulated and 12 were downregulated in the fission/fusion mutant. Of these 30 differentially regulated genes, seven harbored the Pfam domain PF00172 in combination with PF04082 which are characteristic for *Sc*Pdr3 and *Cg*Pdr1 (Supplementary Table S17). Five genes harbored the Pfam domain PF00170 which is characteristic for bZip transcription factors (Supplementary Table S17). Notably, the bZip transcription factor Cap1 of *C. albicans* was previously shown to increase fluconazole tolerance by upregulating expression of the major multidrug transporter Cdr1 (Prasad et al., 2015).

### Construction and Phenotypic Characterization of Conditional ABC Transporter Mutants

**FIGURE 4:**
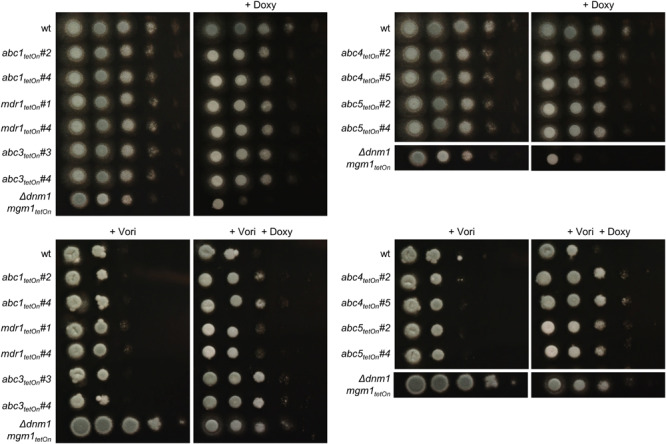
Induction of the conditional *abc1*_tetOn_, *abc3*_tetOn_, *abc4*_tetOn_, and *abc5*_tetOn_ mutants increase azole tolerance of *A. fumigatus*. In a series of 10-fold dilutions derived from a starting suspension of 5 × 10^7^ conidia ml^– 1^ of the indicated strains, aliquots of 3 μl were spotted on Sabouraud agar plates. Two independent conditional mutant clones per ABC transporter are shown. When indicated, medium was supplemented with doxycycline (15 μg ml^– 1^; + Doxy) to induce the Tet-On promoter, or with voriconazole (0.31 μg ml^– 1^; + Vori). Representative images were taken after 24 h (without Vori) or 36 h (+ Vori) incubation at 37°C.

Our data suggested that the overexpression of efflux pumps could be involved in the azole resistance of the fission/fusion mutant. To further investigate the potential role of efflux pumps we constructed conditional mutants of the differentially regulated ABC transporters (Figure 3D and Supplementary Table S14). AFUA_5G06070 (*abc2*) was already denominated *mdr1* in current genome databases, and the respective name was used for consistency. To this end, a well-established doxycycline-inducible Tet-On promoter system was applied (Helmschrott et al., 2013). Under repressive conditions (no doxycycline), this system facilitated significant repression of genes in *A. fumigatus* which resulted in phenotypes similar to those of deletion mutants (Helmschrott et al., 2013; Dichtl et al., 2015; Loiko and Wagener, 2017). Upon induction (e.g., 15 μg ml^–1^ doxycycline) the same promoter enabled overexpression of genes (Helmschrott et al., 2013; Dichtl et al., 2015; Loiko and Wagener, 2017; Fischer et al., 2018). Of each gene the promoter was replaced, essentially as described before (Helmschrott et al., 2013). A technical limitation of this approach is that it cannot be guaranteed that repression and induction of the promoter is sufficient to cause a complete null or overexpression phenotype which may additionally depend on the individual genes. Thus, negative results in this screening do not rule out a role of a tested gene in azole resistance. At least two independent mutant clones were obtained and characterized for each gene. Repression of the Tet-On promoter (no doxycycline) had no significant impact on growth of any of the seven ABC transporter mutants (Figure 4 and Supplementary Figure S2). However, induction of the Tet-On promoter (+ doxycycline) resulted in slightly delayed conidiation compared to wild type, while the overall growth rate of the mycelium was not significantly affected (Figure 4 and Supplementary Figure S2).

To evaluate the impact of the ABC transporters on the azole susceptibility, we performed an initial screening of the obtained mutants with the broth microdilution method (two mutant clones per conditionally regulated gene; Supplementary Table S18). Mutants that appeared more or less susceptible to voriconazole were subsequently confirmed on solid agar. The wild type and conditional fission/fusion mutant Δ*dnm1 mgm1_tetOn_* served as controls. Under repressed conditions all seven ABC transporter mutants exhibited a voriconazole susceptibility similar to wild type (data not shown). Under induced conditions, five of the seven ABC transporter mutants showed slightly altered minimal inhibitory concentrations (MICs) for voriconazole in the broth microdilution assay. Of those, four demonstrated also an increased voriconazole tolerance on solid agar (Figure 4 and Table 1): *abc1*_tetOn_, *abc3*_tetOn_, *abc4*_tetOn_ and *abc5*_tetOn_.

**TABLE 1:** Phenotypes of the conditional ABC transporter mutants.

Mutant	Gene (ORF)	Repressive conditions			Induced conditions		
		Growth	Conidiation	Azole tolerance (MIC)	Growth	Conidiation	Azole tolerance (MIC)
*abc1*_tetOn_	AFUA_3G01400	0	0	0 (0.40)	0	-	+ (0.53)
*mdr1*_tetOn_ (*abc2*_tetOn_)	AFUA_5G06070	0	0	0 (0.40)	0	-	-/0 (0.53)
*abc3*_tetOn_	AFUA_6G08020	0	0	0 (0.40)	0	-	+ (0.53)
*abc4*_tetOn_	AFUA_4G14130	0	0	0 (0.40)	0	-	+ (0.53)
*abc5*_tetOn_	AFUA_6G03080	0	0	0 (0.40)	0	-	+ (0.53)
*abc6*_tetOn_	AFUA_5G10510	0	0	0 (0.40)	0	-	0 (0.40)
*abc7*_tetOn_	AFUA_3G07300	0	0	0 (0.40)	0	-	0 (0.40)

*0, wild type-like; +, increased; -, decreased; MICs (μg ml^–1^) obtained in initial screening with broth microdilution method are shown in brackets; MICs (repressive/induced conditions) of wild type were 0.40/0.40 and of Δdnm1 mgm1_tetOn_ 1.69–2.3/1.27–1.69.*

### Construction and Phenotypic Characterization of Conditional Transcription Factor Mutants

**FIGURE 5:**
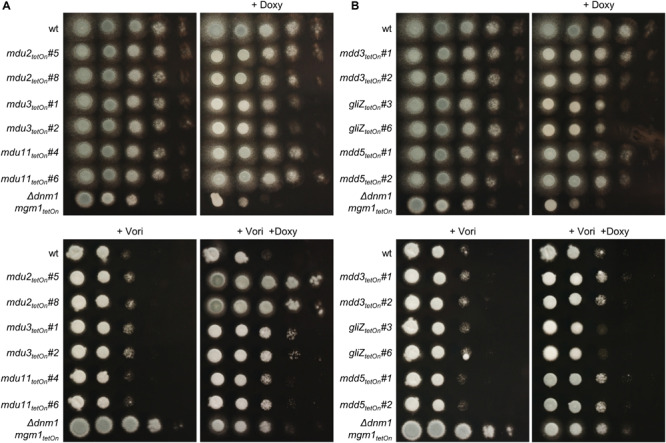
Induction of the conditional *mdu2*_tetOn_, *mdu3*_tetOn_, *mdu11_tetOn_ and of mdd3_tetOn_* and *mdd5*_tetOn_ mutants increase azole tolerance of *A. fumigatus*. In a series of 10-fold dilutions derived from a starting suspension of 5 × 10^7^ conidia ml^– 1^ of the indicated strains, aliquots of 3 μl were spotted on Sabouraud agar plates. Two independent conditional mutant clones of transcription factors that are upregulated **(A)** or downregulated **(B)** in the mitochondrial fission/fusion mutant are shown. When indicated, medium was supplemented with doxycycline (15 μg ml^– 1^; +Doxy) to induce the Tet-On promoter, or with voriconazole (0.41 μg ml^– 1^; +Vori). Representative images were taken after 24 h (without Vori) or 36 h (+Vori) incubation at 37°C.

**FIGURE 6:**
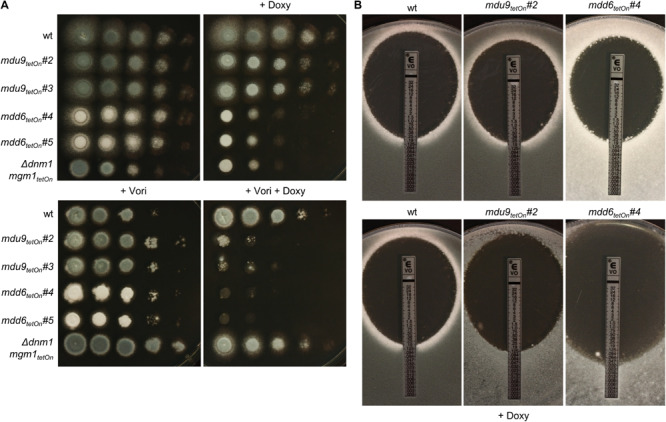
Induction of the conditional *mdu9*_tetOn_ and *mdd6*_tetOn_ mutants result in increased azole susceptibility. **(A)** In a series of 10-fold dilutions derived from a starting suspension of 5 × 10^7^ conidia ml^– 1^ of the indicated strains, aliquots of 3 μl were spotted on Sabouraud agar plates. Two independent conditional mutant clones per transcription factor are shown. When indicated, medium was supplemented with doxycycline (15 μg ml^– 1^; +Doxy) to induce the Tet-On promoter, or with voriconazole (0.23 μg ml^– 1^; + Vori). Representative images were taken after 24 h (without Vori) or 36 h (+Vori) incubation at 37°C. **(B)** 1 × 10^6^ conidia of the indicated strains were spread on Sabouraud agar plates. When indicated (+ Doxy), medium was supplemented with 15 μg ml^– 1^ doxycycline. Voriconazole Etest strips were applied and representative images were taken after 48 h incubation at 37°C.

Next, we used the same approach to study the role of the differentially regulated transcription factors for the azole susceptibility of *A. fumigatus*. Similar to the previously reported autoregulation of the PDR networks in *S. cerevisiae* and *C. glabrata* (Delahodde et al., 1995; Paul et al., 2011), the transcription factors that mediate azole resistance in *A. fumigatus* might show positive or negative regulation of their own expression. Conditional mutants of the 17 mostly affected transcription factors (cut-off log2 = 0.95/log2 = −0.95) were constructed by replacing the promoters of the respective genes with the doxycycline-inducible Tet-On promoter (Figure 3E and Supplementary Table S17). The genes were named mitochondrial dynamics upregulated (mdu) or mitochondrial dynamics downregulated (mdd) for transcription factors that are upregulated or downregulated in the mitochondrial fission/fusion mutant, respectively. AFUA_6G09630 (*mdd4*) was already denominated *gliZ* and AFUA_6G12150 (*mdu8*) *atfD* in current genome databases, and the respective names were used for consistency. Two independent mutant clones were obtained and analyzed for each gene. Of the eleven mdu mutants and six mdd mutants, only the *mdd6*_tetOn_ mutant presented a remarkable phenotype under repressed conditions that was characterized by abolished conidiation (Figures 5, 6A and Supplementary Figure S3). Under induced conditions, the growth rates of the *mdu3*_tetOn_, *mdu9*_tetOn_, *mdd2*_tetOn_, and *gliZ*_tetOn_ (*mdd4*_tetOn_) mutants were moderately and of *mdd6*_tetOn_ strikingly reduced. The mycelium of the *mdd2*_tetOn_ mutant exhibited a brownish-yellow color. Besides this, conidiation of *mdu2*_tetOn_, *mdu3*_tetOn_, *mdu5*_tetOn_, *mdu6*_tetOn_, *mdu7*_tetOn_, *mdu9*_tetOn_, and *mdd3*_tetOn_ were slightly and of *mdd2*_tetOn_, *gliZ*_tetOn_ (*mdd4*_tetOn_), and *mdd6*_tetOn_ strongly reduced (Figures 5, 6A and Supplementary Figure S3).

The azole susceptibility of the mutants was then evaluated using the broth microdilution method (two mutant clones per conditionally regulated gene were tested; Supplementary Table S18). Under repressed conditions, the voriconazole MICs of all mdu and mdd mutants were similar to that of wild type. Under induced conditions, four mdu and four mdd mutants demonstrated slightly altered voriconazole MICs. Interestingly, while the MICs of six mutants were increased, two mutants showed slightly reduced MICs. The decreased azole susceptibilities of the three mdu mutants (*mdu2*_tetOn_, *mdu3*_tetOn_, and *mdu11*_tetOn_) and two of the three mdd mutants (*mdd3*_tetOn_ and *mdd5*_tetOn_) under induced conditions were subsequently confirmed on solid agar (Figure 5 and Table 2). Similar, we could confirm the increased azole susceptibilities of *mdu9*_tetOn_ and *mdd6*_tetOn_ under induced conditions (Figure 6 and Table 2).

**TABLE 2:** Phenotypes of the conditional transcription factor mutants.

Mutant	Gene (orf)	Repressive conditions			Induced conditions		
		Growth	Conidiation	Azole tolerance (MIC)	Growth	Conidiation	Azole tolerance (MIC)
*mdu1*_tetOn_	AFUA_8G07000	0	0	0 (0.40)	0	0	0 (0.40)
*mdu2*_tetOn_	AFUA_1G03800	0	0	0 (0.40)	0	-	+ (0.71)
*mdu3*_tetOn_	AFUA_5G01650	0	0	0 (0.40)	-	-	+ (0.53)
*mdu4*_tetOn_	AFUA_8G07280	0	0	0 (0.40)	0	0	0 (0.40)
*mdu5*_tetOn_	AFUA_2G15340	0	0	0 (0.40)	0	-	0 (0.465)
*mdu6*_tetOn_	AFUA_4G01470	0	0	0 (0.40)	0	–	0 (0.40)
*mdu7*_tetOn_	AFUA_4G00710	0	0	0 (0.40)	0	-	0 (0.465)
*atfD*_tetOn_ (*mdu8*_tetOn_)	AFUA_6G12150	0	0	0 (0.40)	0	0	0 (0.465)
*mdu9*_tetOn_	AFUA_2G09330	0	0	0 (0.40)	-	-	- (0.23–0.3)
*mdu10*_tetOn_	AFUA_1G14860	0	0	0 (0.40)	0	0	0 (0.40)
*mdu11*_tetOn_	AFUA_2G14350	0	0	0 (0.40)	0	0	+ (0.53)
*mdd1*_tetOn_	AFUA_5G14290	0	0	0 (0.40)	0	0	0 (0.40)
*mdd2*_tetOn_	AFUA_1G15910	0	0	0 (0.465)	- (Yellow)	Abolished	0 (0.3–0.4)
*mdd3*_tetOn_	AFUA_6G06535	0	0	0 (0.40)	0	-	+ (0.53)
*gliZ*_tetOn_ (*mdd4*_tetOn_)	AFUA_6G09630	0	0	0 (0.40)	-	–	-/0 (0.53)
*mdd5*_tetOn_	AFUA_6G12160	0	0	0 (0.40)	0	0	+ (0.53)
*mdd6*_tetOn_	AFUA_4G06880	0	Abolished	0 (0.40)	–	–	- (0.17–0.23)

*0, wild type-like; +, increased; -, decreased; –, strongly decreased; MICs (μg ml^–1^) obtained in initial screening with broth microdilution method are shown in brackets; MICs (repressive/induced conditions) of wild type were 0.40/0.40 and of Δdnm1 mgm1_tetOn_ 1.69–2.3/1.27–1.69.*

### Deletion of Individual Transcription Factors or ABC Transporters in the Mitochondrial Fission/Fusion Mutant Does Not Restore Wild Type-Like Azole Susceptibility

**FIGURE 7:**
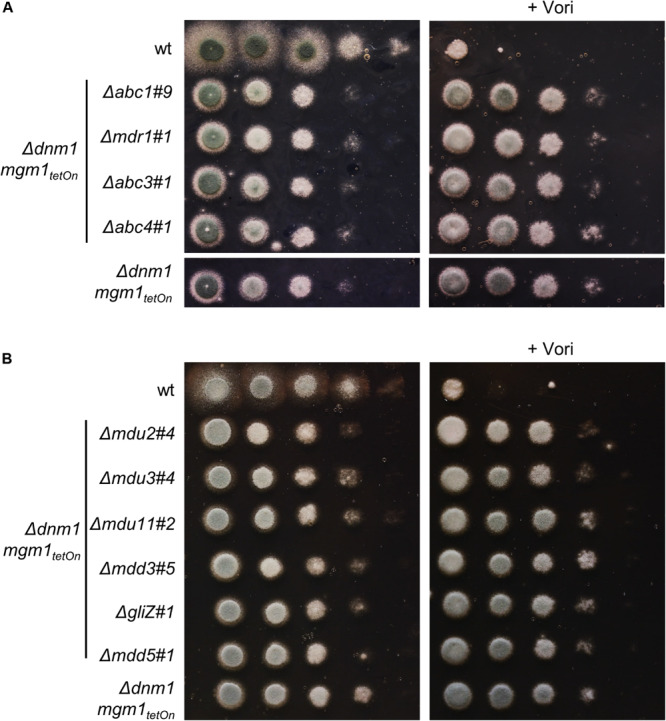
Deletion of genes encoding individual ABC transporters (**A**; *abc1*, *mdr1* (*abc2*), *abc3* and *abc4*) or transcription factors (**B**; *mdu2, mdu3, mdu11, mdd3, gliZ* (*mdd4*) and *mdd5*) in the Δ*dnm1 mgm1_tetOn_* mutant does not affect azole susceptibility. In a series of 10-fold dilutions derived from a starting suspension of 5 × 10^7^ conidia ml^– 1^ of the indicated strains, aliquots of 3 μl were spotted onto Sabouraud (SAB) agar plates. When indicated (+ 0.41 μg ml^– 1^ Vori), medium was supplemented with voriconazole. Plates without voriconazole were incubated for 24 h at 37°C and plates containing voriconazole were incubated for 36 h at 37°C.

We identified four ABC transporters and six transcription factors that demonstrated the ability to increase the azole resistance of *A. fumigatus*. To check whether the increased azole tolerance correlates with an increased expression of the respective genes under induced conditions, the inducibility of two representative conditional mutants was analyzed using quantitative reverse transcription PCR (Supplementary Figure S4). We speculated that one of the identified transporters or transcription factors mediates the increased azole tolerance of the Δ*dnm1 mgm1_tetOn_* mutant, similar to mitochondrial dysfunction-triggered azole resistance via *Sc*Pdr3 and *Cg*Pdr1 in *S. cerevisiae* and *C. glabrata*. We therefore deleted the genes encoding *abc1*, *abc3*, and *abc4* as well as *mdu2*, *mdu3*, *mdu11*, *mdd3*, *gliZ* (*mdd4*), and *mdd5* in the Δ*dnm1 mgm1_tetOn_* mutant. Despite several attempts to delete *abc5* in the Δ*dnm1 mgm1_tetOn_*, we were not able to obtain any mutant clones. Since the *mdr1*_tetOn_ (*abc2*_tetOn_) mutant showed minimally increased azole tolerance under induced conditions in the initial screen with the broth microdilution method, the respective gene (*mdr1*/*abc2*) was also deleted in the fission/fusion mutant. Deletion of these genes did not significantly affect the growth phenotype and azole susceptibility of the Δ*dnm1 mgm1_tetOn_* mutant (Figure 7). However, in contrast to our expectation neither the deletion of the individual ABC transporters nor deletion of individual transcription factors did affect the azole susceptibility of the mitochondrial fission/fusion mutant (Figure 7).

## Discussion

Within our study we aimed on identifying mechanisms responsible for the increased azole tolerance of the mitochondrial fission/fusion mutant Δ*dnm1 mgm1_tetOn_*. To this end, we performed RNA sequencing and analyzed the expression profile of this mutant. This revealed multiple potential explanations for the observed azole tolerance phenotype.

First, the RNA sequencing data indicate an increase in the expression of Cyp51A, the target enzyme of the azole antifungals. Although *A. fumigatus* expresses a second functionally redundant ortholog, Cyp51B (Mellado et al., 2005), mutations in *cyp51A* and in its promoter appear to represent the primary CYP51-related resistance mechanisms in this mold species (Perlin et al., 2017). Indeed, increased expression of *cyp51A* due to a tandem repeat in the promoter region is one of the few known mechanism that mediate azole resistance in *A. fumigatus* (Perlin et al., 2017). In a previous study, resistance of clinical *A. fumigatus* isolates was associated with a four to eightfold increase of *cyp51A* expression (Mellado et al., 2007). Compared to this, the 1,6-fold increase of *cyp51A* expression in the Δ*dnm1 mgm1_tetOn_* mutant appears moderate. However, the azole tolerance of the Δ*dnm1 mgm1_tetOn_* mutant is also moderate (MIC ˜1.27–1.69 μg ml^–1^, Neubauer et al., 2015) when compared to clinical resistant strains that are defined by a MIC > 2 μg ml^–1^ for voriconazole. Taken together, this supports a model where the upregulated *cyp51A* expression could be involved in the increased azole tolerance of the mitochondrial fission/fusion mutant.

A second potential explanation for the increased azole tolerance of the Δ*dnm1 mgm1_tetOn_* mutant is the upregulation of efflux pumps. In fact, we found several ABC transporters (7) and MFS transporters (21) to be upregulated in the mutant. Overexpression of such transporters are known to cause clinically relevant azole resistance of *Candida* species (Morschhäuser, 2016; Prasad et al., 2016). Due to the large number of potentially involved MFS transporters we focused on the seven ABC transporters in this work. With the exception of Mdr1 (Abc2; Fraczek et al., 2013), none of them was previously characterized or linked to azole susceptibility in *A. fumigatus*. Our analysis of the seven conditional ABC transporters mutants under repressed conditions suggests that none of them significantly contributes to the azole tolerance of wild type. In case of Mdr1 (Abc2) this could be interpreted as being contradictory to the results of a previous study. Fraczek and colleagues reported that deletion of *mdr1* (*abc2*; AFUA_5G06070) causes a twofold lower MIC for azoles (Fraczek et al., 2013). Considering this weak impact of *mdr1* (*abc2*) deletion on the MIC (twofold decrease), we speculate that such a phenotype could potentially be masked in our mutant due to a low basal expression of the Tet-On promoter under repressed conditions (*oliC-tetOn*; Helmschrott et al., 2013). The induction of the conditional ABC transporter mutants revealed the general capability of four of the seven ABC transporters to increase the azole tolerance of the mold. Consequently, upregulation of these genes could contribute or even cause the increased azole tolerance of the mitochondrial fission/fusion mutant (Table 1).

Third, we analyzed the capability of 17 most up- or down-regulated transcription factors in the Δ*dnm1 mgm1_tetOn_* mutant to modulate the azole tolerance of *A. fumigatus*. To our knowledge, only *gliZ* (*mdd4*; AFUA_6G09630) and *atfD* (*mdu8*; AFUA_6G12150) have been studied in *A. fumigatus* (Bok et al., 2006; Pereira Silva et al., 2017). *gliZ* was identified to be a major regulator of the gliotoxin biosynthesis gene cluster (Bok et al., 2006). An *atfD* deletion mutant was constructed and characterized by Silva and colleagues very recently (Pereira Silva et al., 2017). However, no specific function could be assigned to *atfD*, except for a moderate role in caspofungin susceptibility (Pereira Silva et al., 2017). Similar to the conditional ABC transporter mutants, none of the conditional *mdd* and *mdu* mutants demonstrated an altered azole susceptibility under repressed conditions. However, induction of the mutants revealed the capability of seven of the 17 transcription factors to affect the azole susceptibility of *A. fumigatus* (Table 2). Interestingly, five of the seven transcription factors increased and two decreased the azole tolerance upon induction of the Tet-On promoter.

**FIGURE 8:**
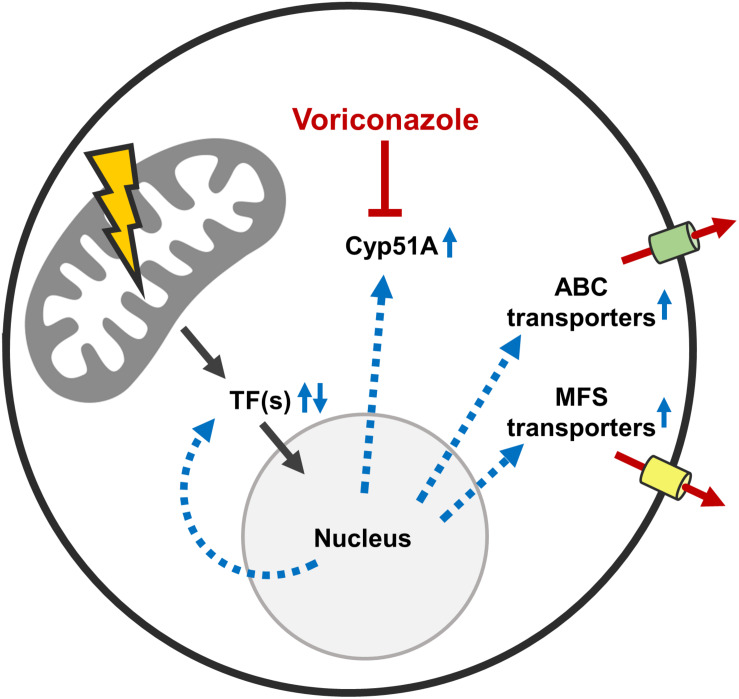
Possible mechanisms that mediate the increased azole tolerance of mitochondrial fission/fusion mutants. Mitochondrial dysfunction triggers altered expression of multiple azole tolerance-affecting genes (black arrows). Among the upregulated genes is the lanosterol 14α-demethylase Cyp51A which is the target enzyme that is inhibited by azole antifungals. Increased Cyp51A expression is a well-characterized azole resistance mechanism. Besides this, multiple transcription factors are up- and downregulated in the Δ*dnm1 mgm1_tetOn_* mutant. At least five of these transcription factors are able to increase and two are able to decrease the azole susceptibility of *A. fumigatus*. In addition, multiple ABC and MFS transporters are upregulated in the Δ*dnm1 mgm1_tetOn_* mutant. At least four of these ABC transporters are able to increase the azole tolerance of the mold. Deletion of individual identified transcription factors and ABC transporters does not diminish the increased azole tolerance of the Δ*dnm1 mgm1_tetOn_* mutant. This suggests that the identified factors are functionally redundant or that other mechanisms are responsible for the azole tolerance of the mitochondrial fission/fusion mutant.

Our transcriptional and experimental data argue for a model where mitochondrial dysfunction triggers the activations of a potentially conserved drug resistance network in *A. fumigatus* (Figure 8). Importantly, mitochondrial dysfunctions other than defects in mitochondrial dynamics were previously shown to also increase the azole tolerance in *A. fumigatus* (Bromley et al., 2016; Kroll et al., 2016; Geißel et al., 2018). A similar response, mediated by a conserved transcription factor which triggers a pleiotropic drug response network, was previously described for baker’s yeast and *C. glabrata* (Traven et al., 2001; Tsai et al., 2006; Gulshan et al., 2008; Ferrari et al., 2009, 2011a,b; Shingu-Vazquez and Traven, 2011; Peng et al., 2012). We therefore tested whether one of the multiple identified ABC transporters or transcription factors with the ability to modulate the azole susceptibility of *A. fumigatus* is specifically responsible for the increased azole tolerance of the mitochondrial fission/fusion mutant. To this end, triple mutants were constructed by deleting each candidate gene, with the exception of *abc5*, in the Δ*dnm1 mgm1_tetOn_* mutant. However, deletion of the individual genes did not significantly affect the increased azole tolerance of the mitochondrial fission/fusion mutant. This indicates that the characterized genes alone are not essential for the observed phenotype. Functional redundancy of the identified factors could explain the absence of an apparent effect on the azole tolerance of the Δ*dnm1 mgm1_tetOn_* mutant. However, to test this hypothesis multiple candidate genes would have to be deleted in the fission/fusion mutant at the same time.

## Data Availability Statement

The datasets generated for this study can be found in the https://www.ncbi.nlm.nih.gov/bioproject/PRJNA627614.

## Author Contributions

JW conceived the study. LS, BG, and JW designed the experiments. LS, BG, and RM performed the experiments. JW and LS wrote the manuscript. All authors analyzed and discussed the data.

## Conflict of Interest

The authors declare that the research was conducted in the absence of any commercial or financial relationships that could be construed as a potential conflict of interest.

## Footnotes

1 https://usegalaxy.org/

2 http://fungidb.org

3 https://www.knime.com

4 http://aspgd.org

5 http://www.yeastgenome.org

6 http://www.candidagenome.org

7 http://pfam.xfam.org/
