# Supplementary material for: Differentially Regulated Transcription Factors and ABC Transporters in a Mitochondrial Dynamics Mutant Can Alter Azole Susceptibility of Aspergillus fumigatus
Source: Front Microbiol. 2020 May 26;11:1017. doi: 10.3389/fmicb.2020.01017 (PMC7264269; doi:10.3389/fmicb.2020.01017)
Supplement: Supplementary file 1 [file Data_Sheet_1.ZIP › Supplementary table 14. Characterized ABC transporters.docx]

**Differentially regulated transcription factors and ABC transporters in a mitochondrial dynamics mutant can alter azole susceptibility of *Aspergillus fumigatus*.**

**Laura Sturm ^1^, Bernadette Geißel ^1^, Johannes Wagener ^1,2,3^***

^1^ Max von Pettenkofer-Institut für Hygiene und Medizinische Mikrobiologie, Medizinische Fakultät, LMU München, 80336 Munich, Germany

^2^ Institut für Hygiene und Mikrobiologie, Julius-Maximilians-Universität Würzburg, 97080 Würzburg, Germany

^3^ National Reference Center for Invasive Fungal Infections (NRZMyk).

* Correspondence: Johannes Wagener, j.wagener@hygiene.uni-wuerzburg.de

| **Gene (orf)** | **Protein name** | **Log 2 (fold change)** | **Classi-fication** | **Orthologues in**  ***S. cerevisiae***  (*, implicated in azole tolerance) | **Orthologues in *C. albicans***  (*, implicated in azole tolerance) | **Function prediction** |
| --- | --- | --- | --- | --- | --- | --- |
| Afu3g01400 | abc1 | 3,10004 | PDR | **YDR011W (SNQ2)*,** **YDR406W (PDR15), YIL013C (PDR11)*, YNR070W (PDR18), YOR011W (AUS1), YOR153W (PDR5)*, YOR328W (PDR10), YPL058C (PDR12)** | **C6_03840C_A (SNQ2),**  **C3_04070C_A (CDR11), C3_04890W_A (CDR2)*, C3_05220W_A (CDR1)*,**  **C1_08070W_A (CDR4)*** | Acting on acid anhydrides; catalyzing transmembrane movement of substances, Putative ABC multidrug transporter |
| Afu5g06070 | abc2, mdr1 | 1,90625 | MDR | YLR188W (MDL1) | CR_02150W_A (MDL1) | ABC multidrug transporter, Xenobiotic-transporting ATPase |
| Afu6g08020 | abc3 | 1,6357 | PDR | **YDR011W (SNQ2)***,  **YDR406W (PDR15), YIL013C (PDR11)*, YNR070W (PDR18), YOR011W (AUS1), YOR153W (PDR5)*, YOR328W (PDR10), YPL058C (PDR12)** | **C6_03840C_A (SNQ2)**, **C1_08070W_A (CDR4)*, C3_04070C_A (CDR11), C3_04890W_A (CDR2)*, C3_05220W_A (CDR1)*** | Acting on acid anhydrides; catalyzing transmembrane movement of substances, Putative ABC transporter with a predicted role in phosphate transport |
| Afu4g14130 | abc4 | 0,934466 | MDR | YKL209C (STE6) | C3_06510C_A (HST6) | Putative MDR1 family ABC transporter, Xenobiotic-transporting ATPase |
| Afu6g03080 | abc5 | 1,00863 | MDR | YPL270W (MDL2) | CR_02150W_A (MDL1) | Putative MDR1 family ABC transporter, Xenobiotic-transporting ATPase |
| Afu5g10510 | abc6 | 0,709005 | MRP/ CFTR | **YLL048C (YBT1)** | C7_02330W_A (YCF1)* | Ortholog(s) have role in secondary metabolite biosynthetic process, Xenobiotic-transporting ATPase |
| Afu3g07300 | abc7 | 0,686112 | PDR | **YDR406W (PDR15),** **YDR011W (SNQ2)*, YIL013C (PDR11)*, YNR070W (PDR18), YOR011W (AUS1), YOR153W (PDR5)*, YOR328W (PDR10), YPL058C (PDR12)** | **C1_08070W_A (CDR4)*, C3_04070C_A (CDR11), C3_04890W_A (CDR2)*, C3_05220W_A (CDR1)*, C6_03840C_A (SNQ2)** | Acting on acid anhydrides; catalyzing transmembrane movement of substances, Has domain(s) with predicted ATP binding, ATPase activity, ATPase activity, coupled to transmembrane movement of substances, nucleoside-triphosphatase activity, nucleotide binding activity and role in transport |

**Supplementary table 14.** Characterized ABC transporters
