# Supplementary material for: Differentially Regulated Transcription Factors and ABC Transporters in a Mitochondrial Dynamics Mutant Can Alter Azole Susceptibility of Aspergillus fumigatus
Source: Front Microbiol. 2020 May 26;11:1017. doi: 10.3389/fmicb.2020.01017 (PMC7264269; doi:10.3389/fmicb.2020.01017)
Supplement: Supplementary file 1 [file Data_Sheet_1.ZIP › Supplementary table 15. Analyzed MFS transporters.docx]

**Differentially regulated transcription factors and ABC transporters in a mitochondrial dynamics mutant can alter azole susceptibility of *Aspergillus fumigatus*.**

**Laura Sturm ^1^, Bernadette Geißel ^1^, Johannes Wagener ^1,2,3^***

^1^ Max von Pettenkofer-Institut für Hygiene und Medizinische Mikrobiologie, Medizinische Fakultät, LMU München, 80336 Munich, Germany

^2^ Institut für Hygiene und Mikrobiologie, Julius-Maximilians-Universität Würzburg, 97080 Würzburg, Germany

^3^ National Reference Center for Invasive Fungal Infections (NRZMyk).

* Correspondence: Johannes Wagener, j.wagener@hygiene.uni-wuerzburg.de

**Supplementary table 15.** Analyzed MFS transporters.

| **Gene (orf)** | **Protein name** | **Log 2 (fold change)** | **Classi-fication** | **Orthologues in**  ***S. cerevisiae***  (*, implicated in azole tolerance) | **Orthologues in**  ***C. albicans***  (*, implicated in azole tolerance) | **Function prediction** |
| --- | --- | --- | --- | --- | --- | --- |
| Afu6g03320 | - | 1,69814 | DHA2 | YKR105C (VBA5) | CR_09390C_A | Has domain(s) with predicted role in transmembrane transport and integral component of membrane localization |
| Afu3g02780 | - | 2,291109 | DHA1 | YLL028W (TPO1)* | C3_03440C_A | Has domain(s) with predicted role in transmembrane transport and integral component of membrane localization |
| Afu2g12710 | - | 1,16841 | - | YOL119C (MCH4) | C2_02580W_A | Has domain(s) with predicted role in transmembrane transport and integral component of membrane localization |
| Afu1g12620 | aflT | 1,01093 | DHA2 | YKR105C (VBA5) | C1_09210C_A | Putative toxin efflux pump |
| Afu4g01140 | - | 1,40188 | DHA1 | YPR156C (TPO3)*, YGR138C (TPO2)* | C1_08790W_A (TPO3) | Putative multidrug resistance protein |
| Afu5g01540 | - | 0,987495 | DHA2 | YKR105C (VBA5) | C1_09210C_A | Has domain(s) with predicted role in transmembrane transport and integral component of membrane localization |
| Afu4g00570 | - | 1,65552 | DHA1 | YLL028W (TPO1)* | C6_04620C_A (NAG4) | Has domain(s) with predicted role in transmembrane transport and integral component of membrane localization |
| Afu2g16860 | - | 1,2486 | DHA1 | YLL028W (TPO1)* | C7_01520W_A (FLU1)* | Putative major facilitator superfamily (MFS) multidrug transporter |
| Afu4g00550 | - | 1,35907 | - | YLR004C (THI73) | C3_03120C_A (DAL5) | Has domain(s) with predicted role in transmembrane transport and integral component of membrane localization |
| Afu5g01630 | - | 0,675907 | DHA1 | YLL028W (TPO1)* | C7_01520W_A (FLU1)* | Ortholog(s) have drug transmembrane transporter activity and role in cellular response to biotic stimulus, drug transmembrane transport, fluconazole transport, peptide transport, spermidine transport |
| Afu3g12900 | hasB | 0,664526 | - | YOR306C (MCH5) | C4_02510W_A | Putative transporter |
| Afu7g04900 | - | 0,936598 | DHA1 | YPR156C (TPO3)* | C3_03440C_A | Has domain(s) with predicted role in transmembrane transport and integral component of membrane localization |
| Afu3g08530 | - | 0,902487 | DHA2 | YKR105C (VBA5) | C5_01270W_A (SGE1) | Has domain(s) with predicted role in transmembrane transport and integral component of membrane localization |
| Afu2g05350 | - | 0,880116 | - | YBR180W (DTR1)* | CR_04620C_A | Putative MFS transporter |
| Afu4g03590 | - | 0,780793 | - | YOL137W (BSC6) | C2_09280C_A | Has domain(s) with predicted role in transmembrane transport and integral component of membrane localization |
| Afu7g06120 | - | 0,602061 | - | YGR260W (TNA1) | C5_03060C_A (TNA1) | Putative transmembrane transporter |
| Afu1g10370 | - | 0,859469 | DHA1 | YLL028W (TPO1)* | C7_01520W_A (FLU1)* | Putative MFS multidrug transporter |
| Afu1g13970 | - | 0,630616 | - | YCR023C | C6_01520W_A | Putative major facilitator superfamily (MFS) transporter |
| Afu2g11580 | - | 1,05573 | DHA1 | YPR156C (TPO3)* | CR_03920C_A (TPO4) | Has domain(s) with predicted role in transmembrane transport and integral component of membrane localization |
| Afu1g11350 | - | 0,947261 | DHA2 | YOR378W (AMF1) | C1_10710C_A | Has domain(s) with predicted role in transmembrane transport and integral component of membrane localization |
| Afu3g09170 | - | 1,22697 | - | YGR260W (TNA1) | CR_01220W_A | Has domain(s) with predicted role in transmembrane transport and integral component of membrane localization |
