# Supplementary material for: Differentially Regulated Transcription Factors and ABC Transporters in a Mitochondrial Dynamics Mutant Can Alter Azole Susceptibility of Aspergillus fumigatus
Source: Front Microbiol. 2020 May 26;11:1017. doi: 10.3389/fmicb.2020.01017 (PMC7264269; doi:10.3389/fmicb.2020.01017)
Supplement: Supplementary file 1 [file Data_Sheet_1.ZIP › Supplementary table 17. Analyzed and characterized transcription factors.docx]

**Differentially regulated transcription factors and ABC transporters in a mitochondrial dynamics mutant can alter azole susceptibility of *Aspergillus fumigatus*.**

**Laura Sturm ^1^, Bernadette Geißel ^1^, Johannes Wagener ^1,2,3^***

^1^ Max von Pettenkofer-Institut für Hygiene und Medizinische Mikrobiologie, Medizinische Fakultät, LMU München, 80336 Munich, Germany

^2^ Institut für Hygiene und Mikrobiologie, Julius-Maximilians-Universität Würzburg, 97080 Würzburg, Germany

^3^ National Reference Center for Invasive Fungal Infections (NRZMyk).

* Correspondence: Johannes Wagener, j.wagener@hygiene.uni-wuerzburg.de

| **Gene (orf)** | **Protein name** | **Log 2 (fold change)** | **Pfam annotation** | | | | | | |
| --- | --- | --- | --- | --- | --- | --- | --- | --- | --- |
|  |  |  | **PF00172** | **PF04082** | **PF00170** | **PF07716** | **PF11951** | **PF05920** | **PF13086/**  **PF13087** |
| Afu8g07280 | mdu4 | 3,56398 | x |  |  |  |  |  |  |
| Afu8g07000 | mdu1 | 2,50092 | x | x |  |  |  |  |  |
| Afu2g15340 | mdu5 | 1,53985 | x |  |  |  |  |  |  |
| Afu4g01470 | mdu6 | 1,47318 | x |  |  |  |  |  |  |
| Afu1g03800 | mdu2 | 1,42847 | x | x |  |  |  |  |  |
| Afu4g00710 | mdu7 | 1,38999 | x |  |  |  |  |  |  |
| Afu6g12150 | mdu8, atfD | 1,16263 |  |  | x |  |  |  |  |
| Afu2g09330 | mdu9 | 1,14618 | x |  |  |  |  |  |  |
| Afu1g14860 | mdu10 | 0,968094 |  |  | x |  |  |  |  |
| Afu2g14350 | mdu11 | 0,959581 |  |  | x |  |  |  |  |
| Afu5g01650 | mdu3 | 0,951499 |  |  | x |  |  |  |  |
| Afu2g04150 | - | 0,931522 |  | x |  |  |  |  |  |
| Afu2g05180 | - | 0,88162 |  |  |  |  |  |  | x |
| Afu3g12180 | - | 0,860504 | x | x |  |  |  |  |  |
| Afu7g06320 | - | 0,825508 | x | x |  |  |  |  |  |
| Afu4g06170 | - | 0,7037 |  |  |  |  |  |  |  |
| Afu3g05760 | - | 0,669588 | x |  |  |  |  |  |  |
| Afu2g00100 | - | 0,649054 | x |  |  |  |  |  |  |
| Afu8g05460 | - | -0,646478 |  |  |  | x |  |  |  |
| Afu2g05380 | - | -0,647657 | x | x |  |  |  |  |  |
| Afu4g10110 | htfA | -0,716771 |  |  |  |  |  | x |  |
| Afu2g14120 | - | -0,753192 |  |  |  |  |  |  |  |
| Afu1g17360 | atfC | -0,763623 |  |  | x |  |  |  |  |
| Afu1g15550 | - | -0,770312 |  |  |  |  |  | x |  |
| Afu4g06880 | mdd6 | -1,09474 |  |  |  |  |  |  |  |
| Afu6g12160 | mdd5 | -1,1073 | x | x |  |  |  |  |  |
| Afu6g09630 | mdd4, gliZ | -1,18389 | x |  |  |  |  |  |  |
| Afu6g06535 | mdd3 | -1,36509 |  |  |  |  |  |  |  |
| Afu1g15910 | mdd2 | -1,47006 | x |  |  |  | x |  |  |
| Afu5g14290 | mdd1 | -1,77606 | x | x |  |  |  |  |  |

**Table 17. Analyzed and characterized transcription factors.**
