# Supplementary material for: Differentially Regulated Transcription Factors and ABC Transporters in a Mitochondrial Dynamics Mutant Can Alter Azole Susceptibility of Aspergillus fumigatus
Source: Front Microbiol. 2020 May 26;11:1017. doi: 10.3389/fmicb.2020.01017 (PMC7264269; doi:10.3389/fmicb.2020.01017)
Supplement: Supplementary file 1 [file Data_Sheet_1.ZIP › Supplementary table 18. MICs obtained for conditional mutants in first screening step.docx]

**Differentially regulated transcription factors and ABC transporters in a mitochondrial dynamics mutant can alter azole susceptibility of *Aspergillus fumigatus*.**

**Laura Sturm ^1^, Bernadette Geißel ^1^, Johannes Wagener ^1,2,3^***

^1^ Max von Pettenkofer-Institut für Hygiene und Medizinische Mikrobiologie, Medizinische Fakultät, LMU München, 80336 Munich, Germany

^2^ Institut für Hygiene und Mikrobiologie, Julius-Maximilians-Universität Würzburg, 97080 Würzburg, Germany

^3^ National Reference Center for Invasive Fungal Infections (NRZMyk).

* Correspondence: Johannes Wagener, j.wagener@hygiene.uni-wuerzburg.de

| **Strain**  **name** | **MIC (µg ml^−1^)** | |
| --- | --- | --- |
|  | **Repressive conditions** | **Induced conditions** |
| AfS35 (wt) | 0.40 | 0.40 |
| *Δdnm1 mgm1_tetOn_* | 1.69-2.3 | 1.27-1.69 |
| *mdu1_tetOn_*#1 | 0.40 | 0.40 |
| *mdu1_tetOn_*#3 | 0.40 | 0.40 |
| *mdu2_tetOn_*#5 | 0.40 | 0.71 |
| *mdu2_tetOn_*#8 | 0.40 | 0.71 |
| *mdu3_tetOn_*#1 | 0.40 | 0.53 |
| *mdu3_tetOn_*#2 | 0.40 | 0.53 |
| *mdu4_tetOn_*#2 | 0.40 | 0.40 |
| *mdu4_tetOn_*#3 | 0.40 | 0.40 |
| *mdu5_tetOn_*#1 | 0.40 | 0.40 |
| *mdu5_tetOn_*#6 | 0.40 | 0.53 |
| *mdu6_tetOn_*#10 | 0.40 | 0.40 |
| *mdu6_tetOn_*#11 | 0.40 | 0.40 |
| *mdu7_tetOn_*#6 | 0.40 | 0.53 |
| *mdu7_tetOn_*#7 | 0.40 | 0.40 |
| *mdu8_tetOn_*#2 | 0.40 | 0.40 |
| *mdu8_tetOn_*#6 | 0.40 | 0.53 |
| *mdu9_tetOn_*#2 | 0.40 | 0.23-0.3 |
| *mdu9_tetOn_*#3 | 0.40 | 0.23-0.3 |
| *mdu10_tetOn_*#2 | 0.40 | 0.40 |
| *mdu10_tetOn_*#8 | 0.40 | 0.40 |
| *mdu11_tetOn_*#2 | 0.40 | 0.53 |
| *mdu11_tetOn_*#4 | 0.40 | 0.53 |
| *mdd1_tetOn_*#3 | 0.40 | 0.40 |
| *mdd1_tetOn_*#4 | 0.40 | 0.40 |
| *mdd2_tetOn_*#2 | 0.53 | 0.30-0.40 |
| *mdd2_tetOn_*#4 | 0.40 | 0.30-0.40 |
| *mdd3_tetOn_*#1 | 0.40 | 0.53 |
| *mdd3_tetOn_*#2 | 0.40 | 0.53 |
| *mdd4_tetOn_*#6 | 0.40 | 0.53 |
| *mdd5_tetOn_*#1 | 0.40 | 0.53 |
| *mdd5_tetOn_*#2 | 0.40 | 0.53 |
| *mdd6_tetOn_*#4 | 0.40 | 0.17-0.23 |
| *mdd6_tetOn_*#5 | 0.40 | 0.17-0.23 |
| *abc1_tetOn_#2* | 0.40 | 0.53 |
| *abc1_tetOn_#4* | 0.40 | 0.53 |
| *abc2_tetOn_#1* | 0.40 | 0.53 |
| *abc2_tetOn_#4* | 0.40 | 0.53 |
| *abc3_tetOn_#3* | 0.40 | 0.53 |
| *abc3_tetOn_#4* | 0.40 | 0.53 |
| *abc4_tetOn_#2* | 0.40 | 0.53 |
| *abc4_tetOn_#5* | 0.40 | 0.53 |
| *abc5_tetOn_#2* | 0.40 | 0.53 |
| *abc5_tetOn_#3* | 0.40 | 0.53 |
| *abc6_tetOn_#15* | 0.40 | 0.40 |
| *abc6_tetOn_#16* | 0.40 | 0.40 |
| *abc7_tetOn_#4* | 0.40 | 0.40 |
| *abc7_tetOn_#5* | 0.40 | 0.40 |
| *abc8_tetOn_#2* | 0.40 | 0.53 |
| *abc8_tetOn_#3* | 0.40 | 0.53 |

**Supplementary table 18.** MICs obtained for conditional mutants in first screening step.
