# Supplementary material for: Differentially Regulated Transcription Factors and ABC Transporters in a Mitochondrial Dynamics Mutant Can Alter Azole Susceptibility of Aspergillus fumigatus
Source: Front Microbiol. 2020 May 26;11:1017. doi: 10.3389/fmicb.2020.01017 (PMC7264269; doi:10.3389/fmicb.2020.01017)

## SUPPLEMENTARY FIGURE LEGENDS

**Supplementary fig. 1. RNA sequencing coverage of *dnm1* and *mgm1* and evaluation of doxycycline-dependent growth of mitochondrial fission, fusion and fission/fusion mutants.** (A) Visualization of RNA sequencing (3 x per strain) results coverage at the *mgm1* and *dnm1* loci, both reverse, of wild type (wt) and Δ*dnm1* $mgm1_{tetOn}$. No sequences obtained from the fission/fusion mutant align to the *dnm1* locus which indicates successful deletion of the gene. Multiple sequences obtained from the fission/fusion mutant align to the conditionally regulated *mgm1* locus, even though the strain was cultured under repressive conditions. Notably, while in the fission/fusion mutant the *mgm1* transcript alignment begins with the start codon of *mgm1* (red arrow, dotted line) because of placement of the conditional Tet-On promoter at this site, the transcript of wild type includes the 5' untranslated region of *mgm1* (blue arrows). (B) Different *A. fumigatus* fusion and fission/fusion mutants were constructed. The mutant used in the present is Δ*dnm1* $mgm1_{tetOn\text{-}pkiA}$ which was constructed by replacing the promoter of *mgm1* in a Δ*dnm1* mutant with the doxycycline-inducible tetOn-pkiA promoter. In parallel, a conditional *mgm1* mutant was constructed by replacing the mgm1 promoter with the tetOn-pkiA promoter ($mgm1_{tetOn\text{-}pkiA}$). The tetOn-pkiA promoter is less leaky and less inducible than the tetOn-gpdA promoter system that was used to construct the $mgm1_{tetOn\text{-}gpdA}$. The $mgm1_{tetOn\text{-}gpdA}$ Δ*dnm1* mutant was constructed by deleting *dnm1* in the conditional $mgm1_{tetOn\text{-}gpdA}$ mutant. In a series of 10-fold dilutions derived from a starting suspension of $5 \times 10^7$ conidia ml-1 of the indicated strains, aliquots of 3 µl were spotted on AMM agar plates. When indicated, medium was supplemented with the indicated amount of doxycycline (µg $ml^{-1}$; +Doxy) to induce the Tet-On promoter. Representative images were taken after 34 h incubation at 37 °C.

**Supplementary fig. 2. Growth phenotypes of seven conditional ABC transporter mutants under repressed and induced conditions.** In a series of 10-fold dilutions derived from a starting suspension of $5 \times 10^7$ conidia $ml^{-1}$ of the indicated strains, aliquots of 3 µl were spotted on Sabouraud agar plates. When indicated, medium was supplemented with doxycycline (15 µg $ml^{-1}$; +Doxy) to induce the Tet-On promoter. Representative images were taken after 24 h incubation at 37 °C.

**Supplementary fig. 3. Growth phenotypes of 17 conditional transcription factor mutants under repressed and induced conditions.** In a series of 10-fold dilutions derived from a starting suspension of $5 \times 10^7$ conidia $ml^{-1}$ of the indicated strains, aliquots of 3 µl were spotted on Sabouraud agar plates. Transcription factors that are upregulated or downregulated in the mitochondrial fission/fusion mutant are depicted in (A) and (B), respectively. When indicated, medium was supplemented with doxycycline (15 µg $ml^{-1}$; +Doxy) to induce the Tet-On promoter. Representative images were taken after 24 h incubation at 37 °C.

**Supplementary fig. 4. Relative expression of *mdd3* and *mdu2* in conditional mutants.** Conidia of the indicated strains were inoculated in triplicates in Sabouraud medium and cultured at 37°C. When indicated (+D), medium was supplemented with doxycycline (15 µg $ml^{-1}$). After 12 h mycelium was harvested, and RNA extracted. Relative expression of *mdd3* (A) and *mdu2* (B) were analyzed with a reverse transcription–qPCR. Statistical significance was calculated using ANOVA with a post-hoc Tukey test. All significant differences are indicated (***, $p \leq 0.001$), all remaining correlations were not significant. Error bars indicate standard deviations.

# Supplementary fig. 1 A

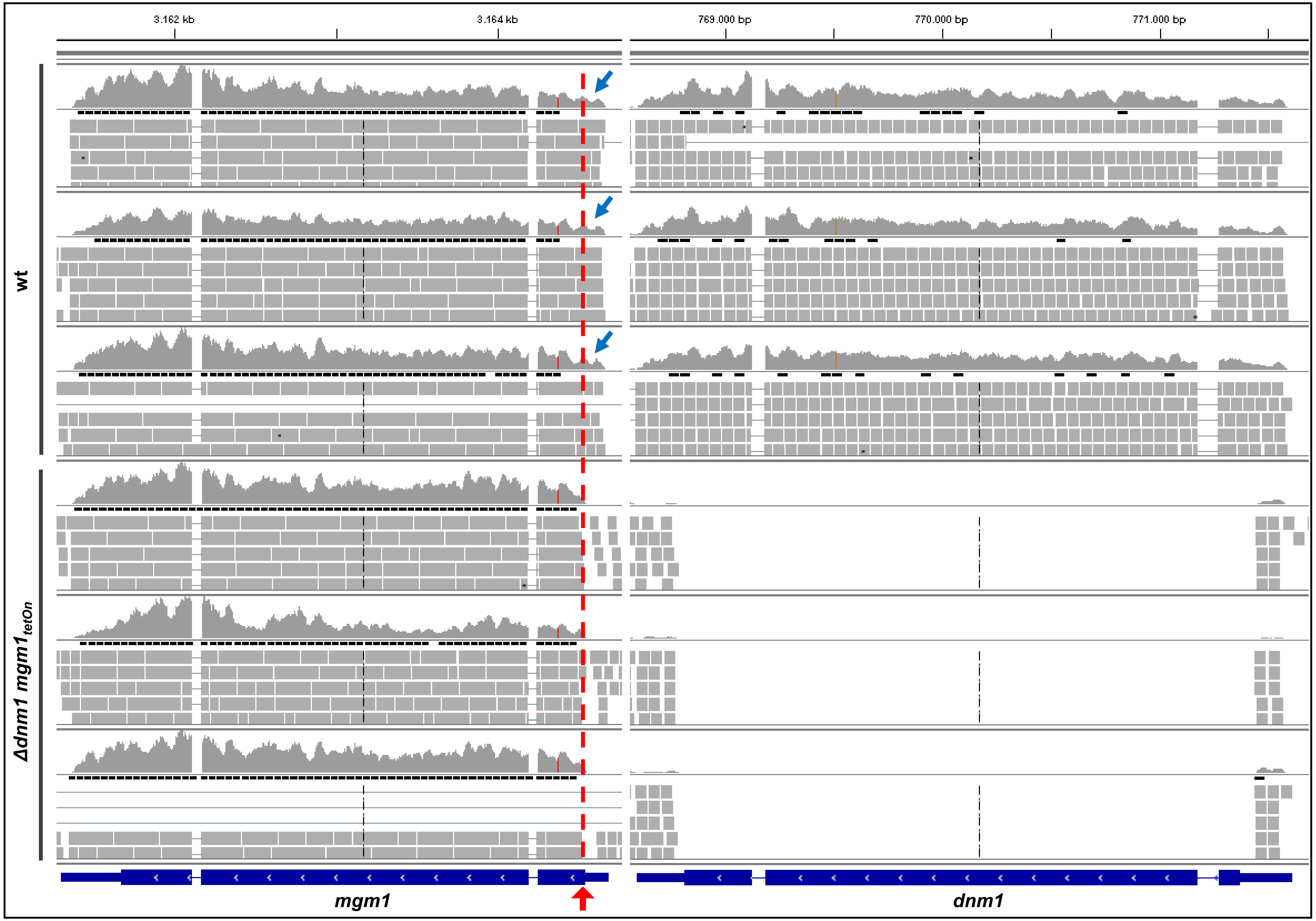

# Supplementary fig. 1 B

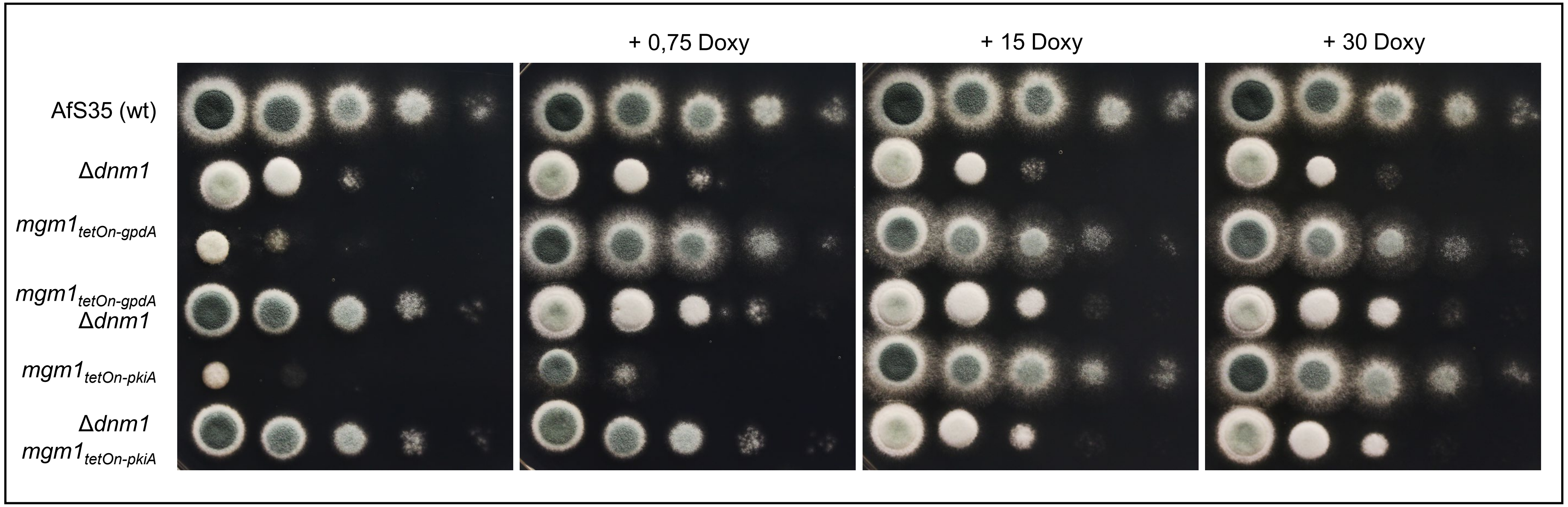

# Supplementary fig. 2

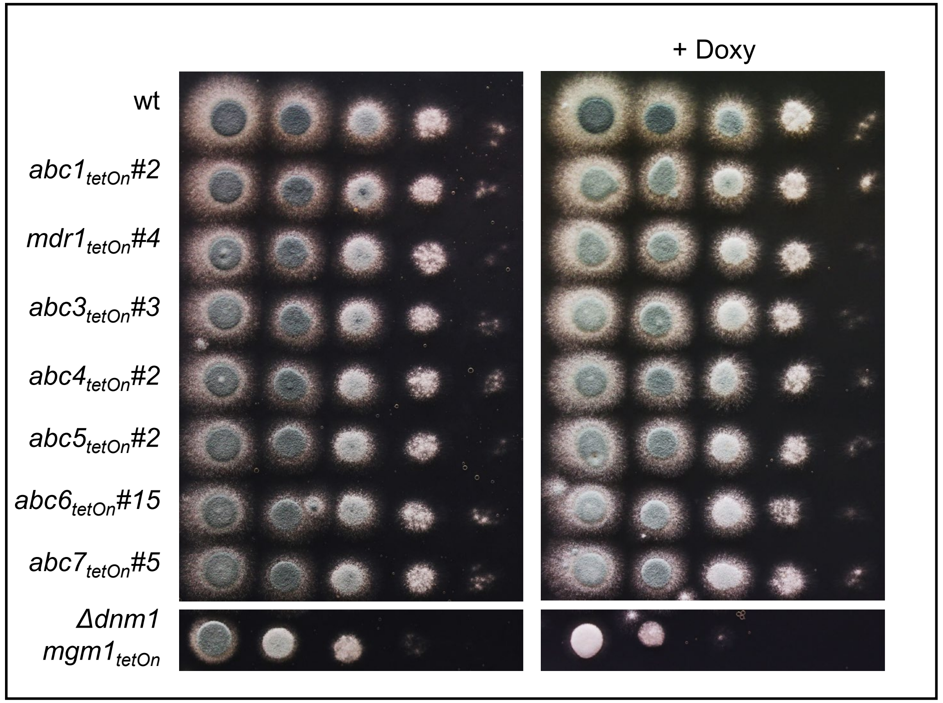

# Supplementary fig. 3

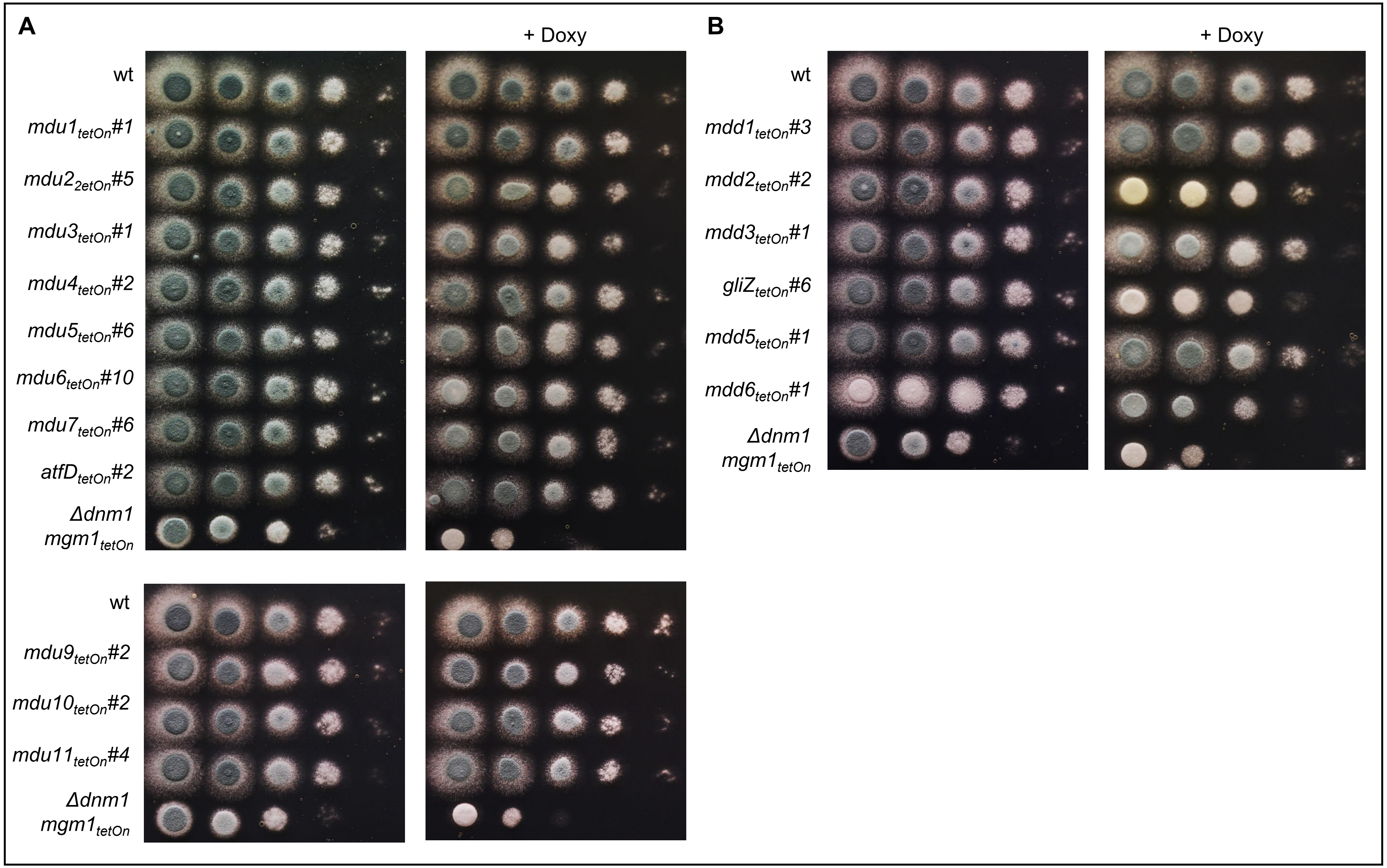

# Supplementary fig. 4

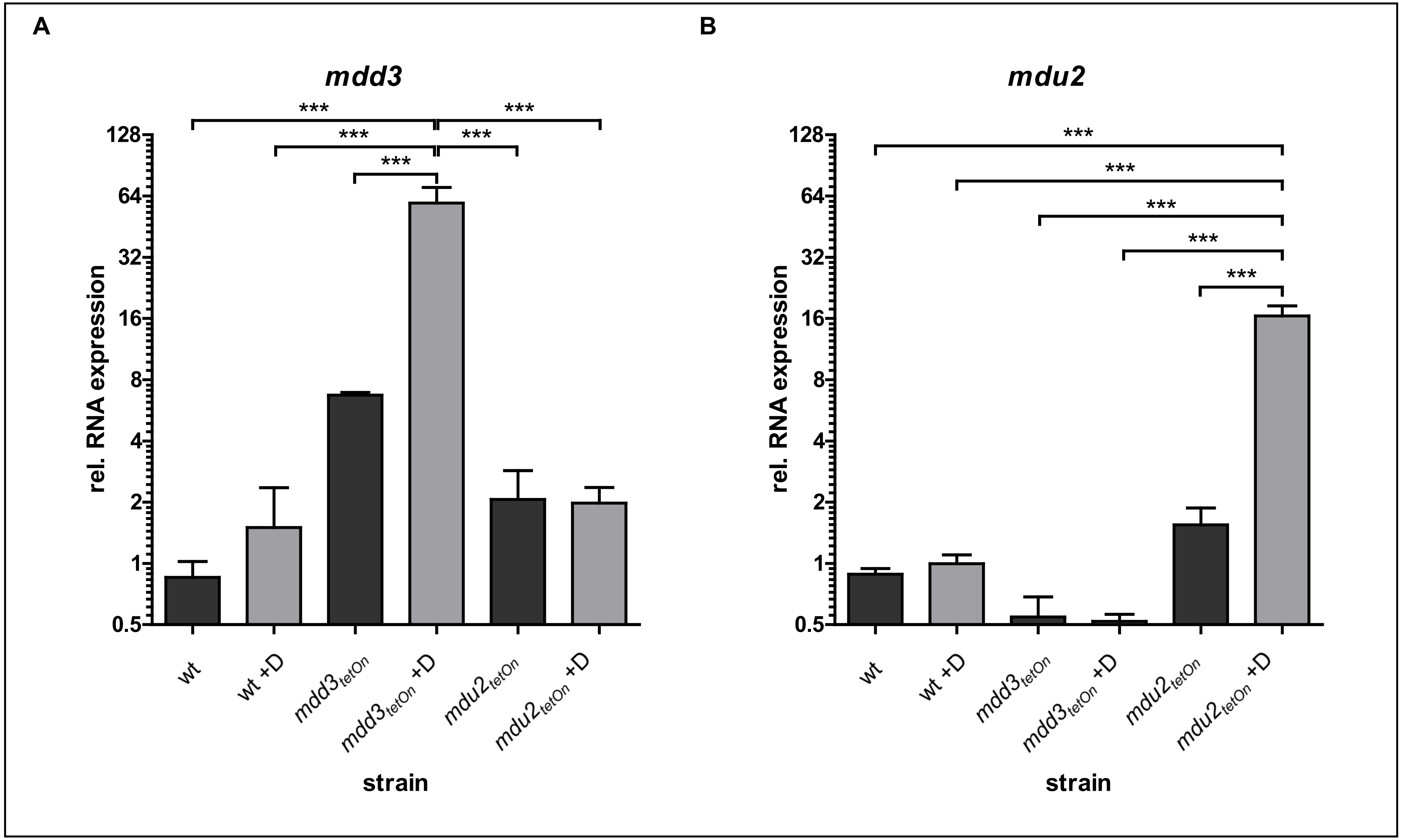

Supplement: Supplementary file 2 [file Data_Sheet_2.PDF]
